# Prevalence of healthy diet and activity behaviours among U.S. Latino preschool children living in an emerging Latino community

**DOI:** 10.1017/jns.2023.50

**Published:** 2023-06-29

**Authors:** Carli A. Liguori, Neil P. Sharma, Patricia I. Documét, Bethany B. Gibbs, Sharon E. Taverno Ross

**Affiliations:** 1 Department of Health and Human Development, University of Pittsburgh, 32 Oak Hill Court, Pittsburgh, PA 15261, USA; 2 Department of Behavioral and Community Health Sciences, University of Pittsburgh, Pittsburgh, PA 15261, USA; 3 Department of Epidemiology and Biostatistics, West Virginia University, Morgantown, WV 26506, USA

**Keywords:** Cross-sectional, Diet, Emerging Latino community, Health, Latino/Hispanic/Latinx, Physical activity, Preschool

## Abstract

This pilot study provides preliminary insights into whether Latino preschool children living in an emerging Latino community (ELC) are meeting recommendations for healthy diet and activity behaviours and whether those behaviours are associated with sociodemographic or home environment variables. Secondary data analysis was conducted utilising cross-sectional baseline survey data from ANDALE Pittsburgh, a home-based intervention study. Measures included parent-reported information on child dietary intake, screen time and the home environment, and objectively measured physical activity and anthropometry. *χ*^2^ and Fischer's exact tests were used to determine associations. The study was conducted in an ELC in western Pennsylvania in the US. Fifty-one Latina mothers (age: 33⋅5 ± 6⋅1 years; 63 % Mexican origin; 86 % low acculturation) and their children (age: 3⋅9 ± 1⋅3 years; 55 % male) 2–5 years of age. On average, children consumed 2⋅25 ± 1⋅44 cups of fruits/vegetables, viewed 98⋅7 ± 74⋅2 min of screen time, accumulated 12⋅9 ± 2⋅9 min/h of total physical activity and consumed 15⋅5 ± 26⋅0 kcals of sugar-sweetened beverages per day. Forty-one percent met the fruit/vegetable recommendation, 54 % met the screen time recommendation, 27 % met the physical activity recommendation and 58 % met the sugary drink recommendation. Country of origin (*P* = 0⋅032) and acculturation (*P* = 0⋅048) were significantly associated with children meeting sugary drink recommendations. No other relationships were significant. The proportion of children in this sample meeting diet and activity recommendations was mixed. More research with larger sample sizes is needed in ELCs to identify effective intervention strategies for improving health behaviours.

## Introduction

The prevalence of childhood obesity is a major public health challenge in the United States that disproportionally affects Latino children. Among preschool children, 16⋅7 % of Latinos are obese, compared to 3⋅5 % of non-Latino whites, 11⋅3 % of non-Latino blacks and 3⋅4 % of non-Latino Asians^(1)^. This is deeply concerning, considering obesity can carry into adulthood, putting individuals at risk for comorbidities across the lifespan^(2)^. The 5-2-1-0 Let's Go! initiative aims to improve health behaviours in children, such as diet and exercise, and has been linked to improved health status later in life^(3)^. With the rapidly growing Latino population in the United States, it is necessary to better understand how healthy lifestyle interventions, such as 5-2-1-0 Let's Go!, impact health outcomes for this expanding population.

The majority of Latinos living in the United States live in established communities with large populations of other Latino people. However, increasingly greater numbers of Latinos are living in emerging Latino communities (ELCs)^(4)^. ELCs are areas with a small but rapidly growing Latino population. The characteristics of ELCs include a scattered Latino population with no concentration in a single neighbourhood or area^(5)^. Those living in ELCs often encounter health, legal and social services that are insufficient and not tailored to their culture or language^(5)^. ELCs differ substantially from established Latino communities, and it is unknown whether patterns in health behaviour observed in established communities will mirror those in ELCs.

Obesity can be treated and prevented by altering lifestyle behaviours such as improving diet quality, increasing physical activity and reducing sedentary behaviour^(6)^. The 5-2-1-0 Let's Go! Initiative, developed in the Barbara Bush Children's Hospital in Portland, Maine, targets health behaviours linked with childhood obesity, specifically those related to physical activity and nutrition^(6–8)^. The daily recommendations of the 5-2-1-0 message include: ‘eat five or more servings of fruits and vegetables’, ‘limit of two hours or less of recreational screen time’, ‘engage in one hour or more of physical activity’ and ‘limit sugary drinks; drink more water and low-fat milk’^(9)^. Encouraging children and families to meet these recommendations promotes the adoption of healthy behaviours that are associated with improved health status and lifelong reductions in chronic disease^(3,9)^. Previous research has identified disparities in adopting these behaviours among Latino preschool children^(10)^. Their influence on Latino children living in ELCs, however, remains unknown and warrants further investigation.

There is evidence that engagement in 5-2-1-0 behaviours varies by sociodemographic and home environmental factors in children. Specifically, lower socio-economic status (e.g. parent education, income, parent employment) is associated with higher screen time^(11)^, increased sugar-sweetened beverage consumption^(12)^, lower fruit and vegetable consumption, and decreased physical activity in children^(13)^. Furthermore, acculturation (i.e. cultural, psychological and behavioural changes that occur in individuals when they come into incessant contact with two or more cultures^(14)^) is associated with poorer health and health behaviours in immigrant youth^(15)^. Other home environmental factors, including parental limit setting, family meals, access to physical activity equipment and having a TV in the bedroom are associated with these 5-2-1-0 behaviours as well as risk for childhood obesity^(16–19)^. Previous research has identified racial/ethnic differences in the prevalence and correlates of 5-2-1-0 behaviours among children and adolescents^(20)^, yet little is known regarding the prevalence and correlates of 5-2-1-0 behaviours in Latino preschool children, particularly those living in ELCs.

The purpose of this pilot study was to provide preliminary insights into whether Latino preschool children living in an ELC are meeting recommendations for healthy diet and activity (5-2-1-0) behaviours and whether those behaviours are associated with key sociodemographic or home environment variables. We hypothesised that in this sample of Latino preschool children: (1) few will meet the recommendations in the 5-2-1-0 message and (2) sociodemographic and home environmental factors will be associated with meeting 5-2-1-0 recommendations.

## Methods

### Setting and participants

The present pilot study was conducted in Allegheny County, Pennsylvania, an ELC^(21)^. The Census Bureau estimated that Latinos comprised 2⋅3 % of the population in Allegheny County in 2019 (27 969 people)^(22)^. The Latino population is spread across the county, with no concentration in any particular neighbourhood. To illustrate the community dispersion, Census Bureau 2018 five-year estimates show that, of the 130 ZIP codes in the county, there are more than 500 Latinos in 12, and more than 1000 in 2; there are no Latinos in only 18 ZIP codes^(23)^.

Participant data was drawn from ANDALE Pittsburgh, a *promotora*-led, home-based child obesity prevention intervention designed to target the home environment to promote a healthy lifestyle in Latino preschool children^(24)^. *Promotoras* (trained community health workers) recruited participants from Allegheny County, Pennsylvania, an ELC. *Promotoras* recruited participants through their social networks, as well as at schools, churches and community gatherings, using flyers and word of mouth. Participating families were eligible if the parent: (1) self-identified as Hispanic/Latino, (2) had at least one child between 2 and 5 years old and (3) spoke either Spanish or English. The present study is a secondary analysis of baseline data collected before participants began the intervention. This study was conducted according to the guidelines laid down in the Declaration of Helsinki and all procedures involving research study participants were approved by the Institutional Review Board at the University of Pittsburgh. Verbal informed consent from parents and verbal assent from children was obtained from all subjects/patients. Verbal consent was witnessed and formally recorded.

### Data collection

A trained, bilingual data collector visited participating families’ homes with the *promotora* to obtain informed consent, deliver an accelerometer, administer a questionnaire and conduct anthropometric measurements on the children.

### Measures

#### Child 5-2-1-0 behaviours

##### Child dietary intake

Parents completed the validated Block Food Screener for Kids 2007 (NutritionQuest, Berkeley, CA)^(25)^. This 15–20-minute screener assesses dietary intake from the past week and has been used previously with Latino children^(26,27)^. NutritionQuest processed the data to estimate specific dietary components. In the current analysis, the variables of interest were total fruit and vegetable servings per day measured in cup equivalents and calories consumed from sugary beverages per day. To determine whether preschool children were meeting recommendations, we modified the guidelines in that children had to: (1) consume 2⋅5 cups of fruits and vegetables and (2) consume no calories from sugary beverages per day in order to be meeting recommendations^(28)^.

##### Child screen time

Parents responded to the question, ‘How much time does your child spend watching TV, playing or working on the internet/computer, OR playing video games per day?’ Responses were measured in minutes per day. Children who engaged in less than 2 h per day of screen time were classified as meeting 5-2-1-0 recommendations.

##### Child physical activity

Children wore an ActiGraph GT3X (Pensacola, FL) accelerometer on an elastic belt on their right hip during a 7-d period before the start of the intervention. Children removed the accelerometer only during sleep hours or water-related activities. The accelerometer collected and stored data in 15-s intervals to capture the sporadic activity patterns typical of young children. Data were reduced using ActiLife version 6 software with non-wear time defined as 60 min of 0 counts^(29,30)^. Data was considered valid if the participants had ≥8 h of wear time on ≥3 d (*n* 22)^(31)^. For our analyses, we modified the 5-2-1-0 physical activity recommendations for preschool children using the Institute of Medicine guidelines^(32)^. Total physical activity (light + moderate + vigorous, using a cutpoint of ≥200 counts/15 s) was averaged over accelerometer wear time as minutes per hour and participants with ≥15 min per hour on average were classified as having met recommendations^(32)^.

#### Home environmental factors

##### Home environment

Using a checklist, parents reported the number of physical activity items available in their home (e.g. basketball hoop/sports goal, wheeled toys, big yard/empty field, etc.). The number of physical activity items available was recorded as low (1–3 items), moderate (4–7 items) or high (8+ items). Parents also responded to the question, ‘During the past week, how many times did all, or most, of your family living in your household eat a meal together?’ The open-ended responses were recorded to low (1–2 meals), moderate (3–6 meals) or high (7 or more meals per week). Finally, parents reported whether their child had a TV in their bedroom (yes/no).

##### Parental limit setting

We examined two questions from the Parenting Strategies for Eating and Activity Scale (PEAS)^(33)^. The first item addressed screen time, ‘I limit the amount of time my child watches TV or videos’ and the second item addressed sugary beverage consumption, ‘I limit the amount of soda/pop my child drinks’. The response options for both items were ‘Strongly Disagree’, ‘Somewhat Disagree’, ‘Somewhat Agree’ and ‘Strongly Agree’. We recorded these responses into disagree (i.e. strongly and somewhat disagree) or agree (i.e. strongly and somewhat agree).

#### Sociodemographic factors

Demographic variables included parent and child age and gender, maternal education, maternal employment status, household income and country of origin. We recoded maternal education into high school or less, and more than high school. Maternal employment status was categorised into full time, part time, stay-at-home caregiver and currently unemployed, but seeking work. Household income was recoded into <$20 000, $20 000–49 999, $50 000–99 999, ≥$100 000 or more or don't know/refused. We recoded country of origin into ‘Mexico’, ‘South America’, ‘Central America’ or ‘Other’.

The 4-item Brief Acculturation Scale for Hispanics asked parents their preferred language in different contexts (reading and speaking; speaking at home; thinking; and speaking with friends). Response options included ‘Only Spanish’, ‘Spanish more than English’, ‘Spanish and English equally’ or ‘English more than Spanish’. Responses were summed and scored with a possible range of 4–20^(34)^. These values were then recoded into low (score 4–9), moderate (score 10–15) and high (score 16–20) acculturation.

Child height and weight were measured in light clothing and without shoes using a mobile stadiometer and digital scale (Seca, CA, USA). Body mass index (BMI) was calculated as body weight (kg)/height (m)^2^; BMI percentiles were based on standardised reference criteria^(35)^. Based on the 2007 Expert Committee recommendations adopted by the Centers for Disease Control^(35)^, children were categorised as normal weight (BMI <85th percentile and >5th percentile), overweight (BMI ≥85th and ≤95th percentile) or obese (>95th percentile).

### Statistical analyses

Descriptive statistics for participant sociodemographic factors, home environment and children meeting 5-2-1-0 recommendations were calculated as either means and standard deviations, or percentages.

We performed *χ*^2^ tests to examine the association between the sociodemographic and home environmental factors with meeting recommendations of the 5-2-1-0 message. If greater than 20 % of the cells had an expected count lower than 5, Fisher's exact test was used. Due to a small sample with valid accelerometer data (*n* 22), we were unable to analyse the relationship between 5-2-1-0 physical activity recommendations and sociodemographic and home environmental factors. All data analyses were performed using IBM SPSS Statistics 25.0 (International Business Machines Corporation, Armonk, NY).

## Results

Participants included fifty-one Latino families (mothers and their preschool-aged child) enrolled in the *promotora*-led, home-based intervention, ANDALE Pittsburgh. The sociodemographic characteristics of participating mothers and their children as well as home environmental factors can be found in Table 1.

**Table 1.** Baseline sociodemographic and home environmental factors of participants from the ANDALE Pittsburgh home-based intervention (*n* 51)

Characteristic	% (*n*) or M	sd
Sociodemographic factors		
Child data		
Child gender, % male	55 % (28)	
Child age, years	3⋅9	1⋅3
Child BMI		
Normal weight (BMI < 85th percentile and ≥5th percentile)	54 % (23)	
Overweight (BMI ≥ 85th percentile and <95th percentile)	20 % (10)	
Obese (BMI ≥ 95th percentile)	26 % (13)	
Parent data		
Maternal age, years	33⋅5	6⋅1
Country of origin		
Mexico	63 % (32)	
South America	20 % (10)	
Central America	14 % (7)	
Other	4 % (2)	
Maternal employment		
Working full time	8 % (4)	
Working part time	16 % (8)	
Stay at home caregiver	71 % (35)	
Currently unemployed, but seeking work	4 % (2)	
Maternal education		
High school or less	55 % (28)	
More than high school	45 % (23)	
Household income		
Less than $20 000	35 % (17)	
$20 000–49 999	20 % (10)	
$50 000–99 999	6 % (3)	
$100 000 or more	6 % (3)	
Don't know/refused	35 % (18)	
Acculturation		
Low (4–9)	86 % (42)	
Moderate (10–15)	10 % (5)	
High (15–20)	4 % (2)	
Home environment factors		
Parent limit setting, % agree		
Television	96 % (48)	
Soda/Pop consumption	88 % (45)	
Number of physical activity equipment items		
Low (1–3 items)	39 % (20)	
Moderate (4–7 items)	37 % (19)	
High (8+ items)	24 % (12)	
Number of family meals per week		
Low (1–2 meals)	12 % (6)	
Moderate (3–6 meals)	32 % (15)	
High (7+ meals)	59 % (30)	
TV in child's bedroom, % yes		
Yes	57 % (29)	
No	43 % (22)	

BMI, body mass index.

Mean child diet, physical activity, and screen time levels and percentage of children meeting recommendations related to the 5-2-1-0 recommendations can be found in Table 2. There were no statistically significant relationships between meeting fruit and vegetable guidelines and any of the sociodemographic and home environmental factors (Table 3).

**Table 2.** Mean child diet, physical activity, and screen time levels and percentage of children meeting recommendations related to the 5-2-1-0 recommendation^a^

Fruit and vegetable consumption (*n* 51)	% (*n*) or M	sd
Fruits and vegetables, cups per day	2⋅25	1⋅44
Did not meet recommendations (<2⋅5 cups/d)	59 % (30)	
Met recommendations (≥2⋅5 cups/d)	41 % (21)	
Screen time (*n* 50)		
Screen time, minutes/day	98⋅7	74⋅2
Did not meet recommendations (≥2 h/d)	46 % (23)	
Met recommendations (<2 h/d)	54 % (27)	
Physical activity (*n* 22)		
Total physical activity, min/h	12⋅9	2⋅9
Did not meet recommendations (<15 min/h total PA)	73 % (16)	
Met recommendations (≥15 min/h total PA)	27 % (6)	
Sugar-sweetened beverage consumption (*n* 51)		
Sugar-sweetened beverages, kcals per day	15⋅5	26⋅0
Did not meet recommendations (>0 kcals/d)	59 % (30)	
Met recommendations (0 kcals/d)	41 % (21)	

M, mean; sd, standard deviation; PA, physical activity.

a Sample size varied based on availability of complete baseline data.

**Table 3.** Association of sociodemographic and home environment factors with children meeting fruit and vegetable recommendations

Characteristic	Not meeting F/V recommendations	Meeting F/V recommendations	*χ*^2^ (*P-*value)
% (*n*)	59 % (30)	41 % (21)	–
Country of origin			4⋅028 (*0*⋅*240*)^†^
Mexico	70 % (21)	52 % (11)	
South America	17 % (5)	28 % (6)	
Central America	13 % (4)	10 % (2)	
Other	0	10 % (2)	
Employment (parent)			3⋅310 (*0*⋅*333*)^†^
Working full time	10 % (3)	5 % (1)	
Working part time	13 % (4)	19 % (4)	
Stay at home caregiver	77 % (23)	67 % (14)	
Currently unemployed, but seeking work	0	10 % (2)	
Education (parent)			2⋅092 (*0*⋅*148)*
High school or less	63 % (19)	43 % (9)	
More than high school	37 % (11)	57 % (12)	
Income			0⋅813 (*0*⋅*973*)^†^
Less than $20 000	33 % (10)	33 % (7)	
$20 000–49 999	17 % (5)	24 % (5)	
$50 000–99 999	7 % (2)	5 % (1)	
$100 000 or more	7 % (2)	5 % (1)	
Don't know/refused	36 % (11)	33 % (7)	
Acculturation			1⋅907 (*0*⋅*494*)^†^
Low (4–9)	90 % (27)	80 % (16)	
Moderate (10–15)	10 % (3)	15 % (3)	
High (16–20)	0	5 % (1)	
Parent limit setting, TV			1⋅457 (*0*⋅*506*)^†^
Agree	93 % (28)	100 % (21)	
Disagree	7 % (2)	0	
Parent limit setting, Soda			0⋅081 (*1*⋅*000*)^†^
Agree	93 % (28)	95 % (20)	
Disagree	7 % (2)	5 % (1)	
Number of physical activity items			1⋅981 (*0*⋅*382*)
Low (1–3 items)	47 % (14)	29 % (6)	
Moderate (4–7 items)	37 % (11)	43 % (9)	
High (8+ items)	16 % (5)	29 % (6)	
Number of family meals per week			0⋅912 (*0*⋅*649*)^†^
Low (1–2 meals)	13 % (4)	10 % (2)	
Moderate (3–6 meals)	33 % (10)	24 % (5)	
High (7+ meals)	53 % (16)	67 % (14)	
TV in child's bedroom			0⋅001 (*0*⋅*973*)
Yes	57 % (17)	57 % (12)	
No	43 % (13)	43 % (9)	

† Denotes use of Fisher's exact test to determine *χ*^2^ and *P*-value.

There were no statistically significant associations between sociodemographic and home environmental factors with children meeting screen time recommendations (Table 4). However, there was a relationship approaching significance (*P* = 0⋅052) between number of family meals per week and children meeting the screen time recommendations: Descriptively, families who ate 3–6 meals together per week had a higher prevalence of children meeting recommendations compared to children with families who ate 1–2 meals or greater than 7 meals per week.

**Table 4.** Association of sociodemographic and home environmental factors with children meeting screen time recommendations

Characteristic	Not meeting screen time recommendations	Meeting screen time recommendations	*χ*^2^ (*P-*value)
% (*n*)	46 % (23)	54 % (27)	–
Country of origin			2⋅910^†^ (*0*⋅*414*)
Mexico	57 % (13)	67 % (18)	
South America	26 % (6)	19 % (5)	
Central America	9 % (2)	15 % (4)	
Other	8 % (2)	0	
Maternal employment			1⋅107^†^ (*0*⋅*888*)
Working full time	4 % (1)	11 % (3)	
Working part time	13 % (3)	15 % (4)	
Stay at home caregiver	78 % (18)	70 % (19)	
Currently unemployed, but seeking work	4 % (1)	4 % (1)	
Maternal education			0⋅057 (*0*⋅*811*)
High school or less	52 % (12)	56 % (15)	
More than high school	48 % (11)	44 % (12)	
Total household income			5⋅398^†^ (*0*⋅*229*)
Less than $20 000	30 % (7)	33 % (9)	
$20 000–49 999	30 % (7)	11 % (3)	
$50 000–99 999	0	11 % (3)	
$100 000 or more	9 % (2)	4 % (1)	
Don't know/refused	30 % (7)	41 % (11)	
Acculturation			1⋅487^†^ (*0*⋅*523*)
Low (4–9)	86 % (19)	85 % (23)	
Moderate (10–15)	9 % (2)	15 % (4)	
High (16–20)	5 % (1)	0	
Parent limit setting, TV			0⋅013^†^ (*0*⋅*708*)
Agree	96 % (22)	96 % (26)	
Disagree	4 % (1)	4 % (1)	
Parent limit setting, Soda			549^†^ (*0*⋅*588*)
Agree	91 % (21)	96 % (26)	
Disagree	9 % (2)	4 % (1)	
Number of physical activity items			4⋅095 (*0*⋅*129*)
Low (1–3 items)	30 % (7)	44 % (12)	
Moderate (4–7 items)	35 % (8)	44 % (12)	
High (8+ items)	35 % (8)	11 % (3)	
Number of family meals per week			5⋅882^†^ (*0*⋅052)
Low (1–2 meals)	13 % (3)	7 % (2)	
Moderate (3–6 meals)	13 % (3)	44 % (12)	
High (7+ meals)	74 % (17)	48 % (13)	
TV in child's bedroom			0⋅911 (*0*⋅*340*)
Yes	65 % (15)	52 % (14)	
No	35 % (8)	48 % (13)	

† Denotes use of Fisher's exact test to determine *χ*^2^ and *P*-value.

Country of origin (*P* = 0⋅032) and acculturation (*P* = 0⋅048) were significantly associated with children meeting sugary drink recommendations (Table 5). Children of Mexican origin, and those with lower acculturation, had a lower prevalence of meeting recommendations compared with children of other countries of origin and higher acculturation scores. There were no other significant relationships between meeting the sugary drink recommendations and sociodemographic and home environmental factors.

**Table 5.** Association of sociodemographic and home environmental factors with children meeting sugary drink recommendations

Characteristic	Not meeting sugary drink recommendations	Meeting sugary drink recommendations	*χ*^2^ (*P-*value)
% (*n*)	59 % (30)	41 % (21)	–
Country of origin			7⋅525^†^ (*0*⋅*032*)
Mexico	86 % (18)	47 % (14)	
South America	10 % (2)	30 % (9)	
Central America	5 % (1)	17 % (5)	
Other	0	7 % (2)	
Maternal employment			1⋅818^†^ (*0*⋅*694*)
Working full time	5 % (1)	10 % (3)	
Working part time	19 % (4)	13 % (4)	
Stay at home caregiver	76 % (16)	70 % (21)	
Currently unemployed, but seeking work	0	7 % (2)	
Maternal education			0⋅072 (*0*⋅*788*)
High school or less	57 % (12)	53 % (16)	
More than high school	43 % (9)	46 % (14)	
Total household income			7⋅378^†^ (*0*⋅*089*)
Less than $20 000	29 % (6)	37 % (11)	
$20 000–49 999	10 % (2)	27 % (8)	
$50 000–99 999	10 % (2)	3 % (1)	
$100 000 or more	0	10 % (3)	
Don't know/refused	52 % (11)	23 % (7)	
Acculturation			5⋅825^†^ (*0*⋅*047*)
Low (4–9)	100 % (21)	76 % (22)	
Moderate (10–15)	0	21 % (6)	
High (16–20)	0	3 % (1)	
Parent limit setting, TV			1⋅457^†^ (*0*⋅*506*)
Agree	100 % (21)	93 % (28)	
Disagree	0	7 % (2)	
Parent limit setting, Soda			0⋅855^†^ (*0*⋅*561*)
Agree	91 % (19)	97 % (29)	
Disagree	9 % (2)	3 % (1)	
Number of physical activity items			1⋅532 (*0*⋅*465*)
Low (1–3 items)	48 % (10)	33 % (10)	
Moderate (4–7 items)	38 % (8)	40 % (12)	
High (8+ items)	14 % (3)	27 % (8)	
Number of family meals per week			354^†^ (*0*⋅*921*)
Low (1–2 meals)	14 % (3)	10 % (3)	
Moderate (3–6 meals)	29 % (6)	30 % (9)	
High (7+ meals)	57 % (12)	60 % (18)	
TV in child's bedroom			0⋅370 (*0*⋅*543*)
Yes	62 % (13)	53 % (16)	
No	38 % (8)	47 % (14)	

† Denotes use of Fisher's exact test to determine *χ*^2^ and *P*-value.

## Discussion

The achievement of the 5-2-1-0 recommendations in this sample of preschool Latino children living in an ELC was mixed. Descriptively, 41 % of participants met the fruit and vegetable recommendation, 54 % met the screen time recommendation, 27 % met the physical activity recommendation and 58 % met the SSB recommendation.

Previous studies conducted in children 6–11 years old have observed that less than half of Latino children met even one of the 5-2-1-0 recommendations, however these investigations have not included younger children nor assessed determinants of meeting these recommendations in the preschool age group^(20)^. We found that fewer children of Mexican origin were meeting recommendations compared with children of other origins. Mexico is the second largest consumer of soft drinks in the world^(36)^, and a report from the Mexican government found that preschool children consumed 27⋅8 % of their energy from caloric beverages (e.g. whole milk, juices, soft drinks)^(37)^. Mexican immigrants and their descendants represent the largest national-origin group among Latinos living in the US^(38)^. Our data suggests that meeting sugary beverage recommendations may differ by country of origin. This supports the assertion that Latinos are a heterogeneous group with significant differences in dietary and health behaviours among subgroups. Strategic, culturally tailored messaging as a means of decreasing sugar-sweetened beverage consumption is warranted^(39)^.

In our sample, preschool children with mothers who had lower language acculturation were less likely to meet sugary beverage recommendations. These findings contrast to a previous study by Wiley *et al.* which found that preschool children with higher acculturated Latina mothers consumed more unhealthy foods and beverages (such as doughnuts, cookies, cakes, processed foods and sugar-sweetened beverages)^(28)^. Similar associations between mothers’ level of acculturation and child weight status have been observed among children of Mexican immigrants in California's farmworker communities^(40)^. Subsequent research has further supported the negative impact of parent and child acculturation on both diet and physical activity^(41,42)^. However, the majority of these samples consisted of mothers and children from established Latino populations, while our sample was drawn from an ELC in Southwestern Pennsylvania. More research is needed to examine these relationships in both established Latino communities and ELCs, as it is possible that these relationships look different in Latino families living in emerging communities.

There is evidence that the frequency of family meals is lower in Latino immigrant families compared to native Latino families potentially due to acculturative stress, lack of social support and pressure to engage in ‘American’ eating habits^(43)^. Nearly 60 % of our sample reported eating 7+ family meals per week. While not statistically significant, children who ate 3–6 family meals per week together were more likely to meet screen time recommendations compared to those who ate 1–2 and 7 or more family meals per week. According to prior studies, TV viewing during family meals increases the risk for childhood obesity and is associated with insufficient consumption of fruit and vegetables and greater likelihood of the consumption of obesity-promoting foods^(44)^. Future studies with larger sample sizes should explore these complex, and likely culturally derived, relationships.

Although the literature supports several sociodemographic and home environmental factors as correlates for children's 5-2-1-0 behaviours, this was generally not supported in this sample of Latino parents and their preschool-aged children. Lower socio-economic status (e.g. parent education, income, parent employment) has been associated with increased screen time^(19)^ and consumption of sugar-sweetened beverages^(12)^, as well as lower fruit and vegetable consumption and physical activity in children^(13)^; however, these results were not confirmed in this sample of Latino children. Small sample size and low variability on some of these measures may have contributed to the null finding. It is also possible that these relationships look differently in Latino families living in emerging communities; as such, future research into these health behaviours is warranted.

For Latino preschool children, parental limit setting has been observed to have both a negative and positive effect on children's healthy eating habits^(45,46)^. In the present study, the majority of parents agreed that they set limits on both their preschool-aged child's TV viewing (96 %) and soda/pop consumption (88 %); however, this did not translate into higher levels of children meeting screen time or sugary drink recommendations. Furthermore, other home environmental factors associated with obesity in preschool children, such as a TV in the bedroom and greater accessibility to physical activity equipment^(18,19)^, were not associated in our study with meeting recommendations. Again, this raises questions surrounding the determinants of healthy (5-2-1-0) behaviours in this population and suggests that these relationships could differ for Latino families living in an ELC, where social support and access to linguistically and culturally appropriate resources are low^(5)^.

### Strengths and limitations

Very little is known about health behaviours among Latino children living in ELCs. This pilot study provides valuable, preliminary insight into dietary habits, physical activity, sociodemographic and home environmental factors in this population. Most research observing the 5-2-1-0 message and meeting guidelines to date has been in non-Latino preschool children^(47,48)^. The sample for this study comprised participants living in an ELC, while most of the published literature examining the determinants of health behaviours in Latino children used samples drawn from established Latino communities^(10,28,40–42)^. While not population-based, the sample was representative of the country of origin and socio-economic background of the Latino population living in Allegheny County, Pennsylvania.

This small pilot study only included fifty-one participants. This likely limited our precision of estimates and our ability to observe statistically significant differences in meeting recommendations across sociodemographic or home environment characteristics. This limitation is greater for the accelerometry data (*n* 22). In addition, the cross-sectional study design precludes us from establishing causality. The language-based acculturation measure was significantly related to whether children met sugary drink recommendations. More comprehensive measures of acculturation could elicit different relationships. Lastly, the Block Kids Food Screener may have low validity for certain food groups^(49)^, and because most data were parent-reported, there is the potential for overreporting healthy behaviours (e.g. limit setting, family meals, higher fruit and vegetable consumption) and underreporting unhealthy behaviours (e.g. screen time, sugary beverage consumption)^(50)^.

## Conclusions

In conclusion, the current findings suggest that this sample of Latino preschool children living in an ELC only partially met 5-2-1-0 dietary and activity recommendations. The prevalence of meeting recommendations appears to be lower than among children in other racial/ethnic groups, putting Latino preschool children at greater risk for childhood obesity, and exacerbating the obesity disparity longitudinally, and researchers should consider acculturation and country of origin to more effectively support 5-2-1-0 behaviours among Latino families. The majority of what we know about Latino preschool children included samples from established Latino communities, supporting the need to examine these relationships in emerging communities, as these are becoming more prevalent with the growing Latino population in the US. This study should be replicated with a larger sample size to better understand the needs of these dynamic communities. In summary, these findings suggest the need for longitudinal research in ELCs to understand the determinants of obesity in preschool children and to identify culturally sensitive intervention approaches.
